# Screening of glucose-6-phosphate dehydrogenase deficiency in a cohort of 215,137 newborns: an epidemiological and pathogenic variant spectrum study in Yueyang, China

**DOI:** 10.3389/fgene.2026.1810076

**Published:** 2026-06-25

**Authors:** Na Cao, Danchen Xie, Xia Zhou, Hongxiang Mu, Yan Yuan, Li Wan, Ang Sun, Cexun Hu

**Affiliations:** 1 Department of Hematology, Yueyang People’s Hospital, Yueyang, China; 2 Yueyang Hospital Affiliated to Hunan Normal University, Yueyang, China; 3 Department of Medical Genetics, Yueyang Maternal and Child Health-Care Hospital, Yueyang, China; 4 Yueyang Key Laboratory of Birth Defects Prevention and Control, Yueyang, China

**Keywords:** cut-off value, G6PD, glucose-6-phosphate dehydrogenase, newborn screening, pathogenic variant

## Abstract

**Background:**

Glucose-6-phosphate dehydrogenase (G6PD) deficiency is a prevalent inherited metabolic disorder, affecting approximately 500 million individuals worldwide. Current neonatal screening protocols necessitate continuous refinement of cut-off values based on gestational age, birth weight, and seasonal variation. Genetic characterization of G6PD deficiency has become a critical component of newborn screening programs; however, the spectrum of pathogenic variants in the Yueyang region remains elusive.

**Methods:**

G6PD enzymatic activity was quantitatively assessed using fluorescence analysis of dried blood spot samples. Infants with abnormal results were recalled for targeted genetic testing focusing on known hotspot mutations.

**Results:**

A total of 215,137 newborns in Yueyang city underwent screening for G6PD deficiency, yielding an estimated overall birth prevalence of 4.77‰. Gestational age showed a consistent, independent negative correlation with G6PD enzymatic activity. The optimal cut-off value for male newborns was adjusted from 2.6 U/g Hb to 3.2 U/g Hb during winter. In total, 336 neonates were confirmed through the genetic diagnosis of G6PD deficiency. The most frequent pathogenic variant was c.1376G>T, accounting for 31.85% of cases, followed by c.1388G>A (22.92%), c.1311C>T (14.88%), c.95A>G (9.82%), c.1024C>T (8.33%), and c.871G>A (6.85%). The c.1376G>T mutation was associated with the greatest reduction in enzymatic activity, with median levels of 0.8 U/g Hb in males and 3.54 U/g Hb in females.

**Conclusion:**

Stratified by gestational age and seasonal variation, cut-off value optimization is essential for ensuring the efficacy of neonatal G6PD deficiency screening. This study primarily focuses on identifying asymptomatic G6PD deficiency and preventing severe hyperbilirubinemia and neurologic damage. Early screening, timely identification, and standardized follow-up should be prioritized to facilitate the implementation of eugenics-related public health strategies.

## 1 Introduction

Glucose-6-phosphate dehydrogenase (G6PD) deficiency is a prevalent inherited metabolic disorder in the neonatal period (Lee et al., 2022; El et al., 2024). It represents the most common enzymopathy worldwide, affecting approximately 500 million individuals (Maceda et al., 2024). G6PD plays a crucial role in safeguarding cells against oxidative stress. Upon exposure to oxidative stimuli—such as certain foods, medications, or infections—affected individuals may develop acute hemolysis, potentially impairing normal development and posing life-threatening risks (TeSlaa et al., 2023; Leung-Pineda et al., 2023). Males are generally more severely affected due to hemizygosity of the X chromosome, whereas females may be heterozygous carriers with mild to moderate enzyme deficiency (Errigo et al., 2023; Aung et al., 2023). Clinical manifestations vary considerably (Lee et al., 2022; Chamchoy et al., 2023); mild cases may be asymptomatic, whereas severe cases often present with neonatal jaundice and hemolytic anemia (Boonpeng et al., 2024). G6PD deficiency is an X-linked, incompletely dominant disorder caused by mutations in the G6PD gene, resulting in reduced enzyme activity or structural abnormalities (Jinfu et al., 2024). To date, more than 200 G6PD gene mutations have been reported globally, primarily single-nucleotide variants. In China, over 35 mutation types have been identified, with the c.1388G>A, c.1376G>T, and c.95A>G alleles being the most prevalent, collectively accounting for approximately 95% of cases (Geck et al., 2023). Notably, the pathogenic variant spectrum exhibits regional heterogeneity (Zailani et al., 2025), suggesting that integrating local mutational profiles into diagnostic algorithms may improve diagnostic accuracy and cost-effectiveness. However, the specific G6PD pathogenic variant spectrum in the Yueyang region remains incompletely characterized.

Given that G6PD deficiency is an inherited metabolic disorder, avoiding exposure to high-risk factors that may induce acute hemolysis constitutes the optimal management strategy for asymptomatic affected children (Leung-Pineda et al., 2023). Therefore, newborn screening (NBS) is pivotal for early identification (Tan et al., 2024), enabling patients to be informed of relevant risks at an early stage. Since 2014, a newborn screening program has been implemented in northern Hunan. Prior to 2022, infants with suspected positive screening results were recalled for G6PD/6PGD enzymatic ratio testing to confirm the diagnosis of G6PD deficiency. However, it has a notable limitation in detecting heterozygous females. In 2022, G6PD deficiency was incorporated into the national free genetic diagnostic testing program, resulting in increased identification of affected neonates. In recent years, some institutions have established cut-off values for G6PD activity based on gestational age, sex, and birth weight (Zailani et al., 2025; Li et al., 2024; Zhang et al., 2025); however, these values vary across studies, likely due to regional differences. Moreover, the influence of season and ambient temperature on G6PD activity remains unclear. Consequently, laboratory-specific optimization of cut-off values is warranted.

This study aims to determine the birth prevalence of G6PD deficiency and systematically evaluate the key factors influencing the cut-off values in the Yueyang region. Specifically, the G6PD pathogenic variant spectrum is comprehensively summarized, along with a statistical analysis of the correlation between the pathogenic variants and G6PD enzyme activity. The findings are expected to clarify the epidemiological characteristics of G6PD deficiency and provide a robust theoretical foundation for birth defect prevention and control in northern Hunan.

## 2 Materials and methodology

### 2.1 Basic information

From January 2019 to November 2025, 215,137 newborn screening procedures were performed at the Neonatal Screening (NBS) Center of the Key Laboratory of Birth Defects Prevention and Control in Yueyang City. Written informed consent was obtained from all legal guardians of the participants.

### 2.2 Sample collection requirements and methods

The center oversees neonatal screening in three districts and six counties: Yueyanglou District, Yunxi District, Junshan District, Yueyang County, Huarong County, Xiangyin County, Pingjiang County, Miluo City, and Linxiang City. All delivery institutions within the designated region strictly adhered to the “Technical Specifications for Neonatal Disease Screening” issued by the Ministry of Health in 2010 and the requirements set forth by the Maternal and Child Health Department of the Hunan Provincial Health Commission. Blood samples were collected from the medial or lateral edge of the heel after 72 h of birth, provided the infant had received routine breastfeeding for at least eight feedings. Four blood drops were spotted onto specialized filter paper by trained medical personnel, with each spot measuring approximately 8 mm in diameter. The blood spots were air-dried at room temperature, placed in sealed plastic bags, and stored at 2 °C–8 °C. Samples were transported to the screening center within five working days via internal logistics.

### 2.3 Neonatal G6PD activity screening

**FIGURE 1:**
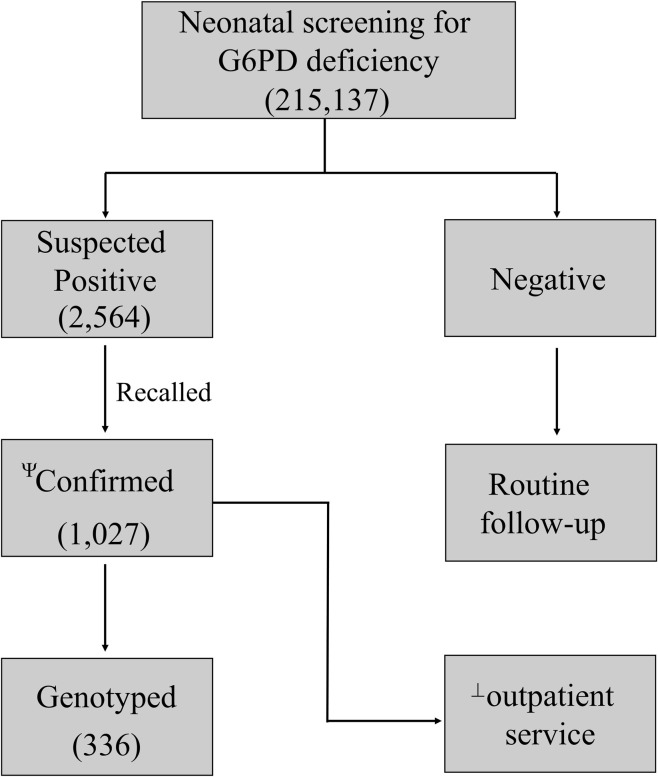
The flow diagram of newborn screening for G6PD deficiency. ^Ψ^Among the 1,027 confirmed cases, genetic mutation results from a total of 336 cases were utilized for data analysis. ^⊥^Outpatient services primarily provide detailed precautions, including a list of dietary restrictions and medications requiring caution.

G6PD enzyme activity was quantitatively assessed using fluorescence analysis from 3 mm dried blood spots (DBS). Reagents and standards were provided by Guangzhou Fenghua Technology Co., Ltd. Quality control materials included both low- and high-concentration levels. Assays were performed using a fully automated time-resolved fluorescence immunoassay analyzer (AutoTRFIA-4) and an automated punching instrument (ABS220). Quality control procedures were conducted in strict accordance with the manufacturer’s instructions. Analytical accuracy was further verified through external quality assessment programs, quarterly organized by the National Clinical Laboratory Center of the Ministry of Health. A sample was classified as screen-positive based on predefined sex-specific cut-off values (male: ≤2.6 U/g Hb; female: ≤4.1 U/g Hb). Suspected positive cases were recalled for repeat DBS sampling and the G6PD/6PGD ratio assay or genetic testing for hotspot mutations. Upon confirmation, the patient was referred to receive outpatient services. Cases were recorded in the case management ledger. Timely follow-up and detailed precautionary guidance were provided, including a list of dietary restrictions and medications to be used with caution. The workflow is shown in Figure 1.

### 2.4 Genetic diagnosis

DBS samples were punched using an automated perforator. In individuals with suspected positive screening results who returned for follow-up, pathogenic variants were analyzed via polymerase chain reaction (PCR) combined with flow-through hybridization, enabling qualitative detection of 10 known G6PD mutations: c.1360C > T, c.1376G > T, c.1388G > A, c.871G > A, c.1024C > T, c.95A > G, c.392G > T, c.487G > A, c.592C > T, and c.1311C > T. If genotype results were inconsistent with the clinical phenotype, further sequencing was performance.

### 2.5 Grouping criteria

Gestational age (GA): preterm birth group<37 weeks; full-term group: 37–41^+6^ weeks; post-term pregnancy group≥42 weeks.

Birth weight: low weight<2,500 g; normal birth weight: 2,500–3,999 g; macrosomia≥4,000 g.

### 2.6 Ethics statement

The studies involving human participants were approved by the Ethics Review Committee of Yueyang Prenatal Diagnosis Center (No. 20251205007) and conducted in accordance with the institutional requirements. Written informed consent for participation was obtained from the legal guardians or next of kin of all participants.

### 2.7 Statistical analysis

Statistical analysis was conducted using GraphPad Prism 8 and SPSS 27.0 software. A non-parametric Kruskal–Wallis test was applied to evaluate differences among the three groups. Post hoc comparisons were performed using Dunn’s multiple comparisons test to identify specific group differences. Receiver operating characteristic (ROC) curve analyses were conducted in MedCalc to assess diagnostic performance. Screening performance was quantified by the area under the curve (AUC), sensitivity, specificity, and Youden index. An AUC≥0.9 was considered indicative of extremely high diagnostic accuracy. A p-value <0.05 was regarded as statistically significant.

## 3 Results

### 3.1 Screening data analysis

**FIGURE 2:**
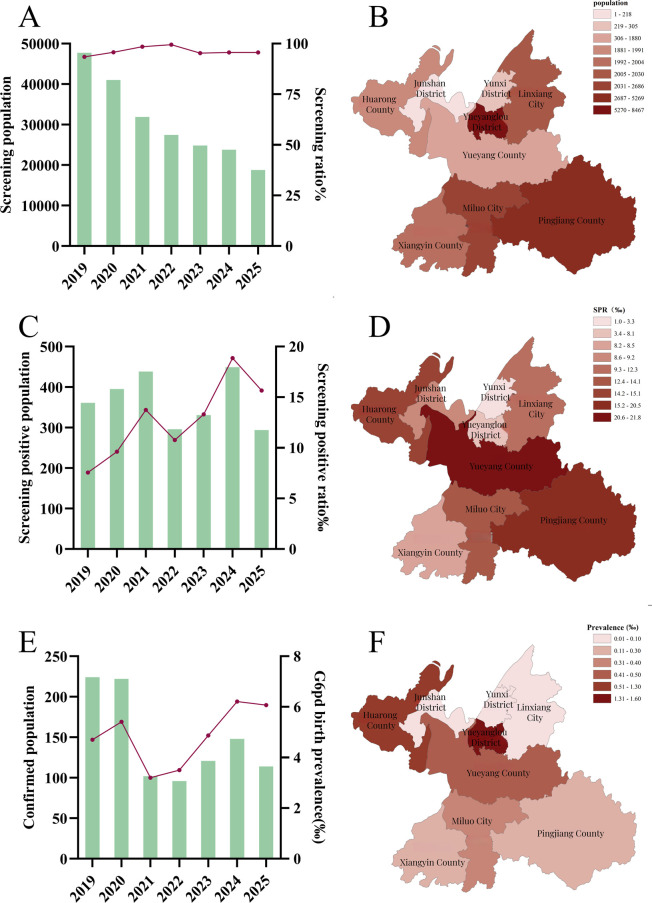
Screening results of G6PD deficiency in Yueyang from 2019 to 2025. Panels **(A,C,E)** depict the overall screening status during this period. **(A)** The screening population (green bar) markedly declined each year, from 47,720 in 2019 to 18,784 in 2025. The screening ratio (red point) remained stable at approximately 95%. **(C)** The number of positive screening results (green bar) exhibited considerable fluctuation, with a notable increase in recent years, likely attributable to advancements in screening technology and logistics. The screening positive ratio (red point) peaked in 2024 at approximately 18.85‰. **(E)** The number of confirmed cases (green bar) decreased by roughly one-third in recent years. In contrast, the birth prevalence of G6PD deficiency (red point) rose sharply, likely due to the introduction of free genetic testing. Panels **(B,D,F)** illustrate the detailed screening data from the Yueyang area. In total, 23,815 newborns were screened for G6PD deficiency in 2024. In Yueyanglou District, both the screening population and the incidence of G6PD deficiency were the highest, whereas the SPR was relatively lower. The highest number of positive screenings occurred in Yueyang County. SR: Screening Ratio; SPR: Screening Positive Ratio.

Over a 7-year period, 215,137 newborns underwent NBS for G6PD deficiency in the Yueyang region (Figure 2). The number of screenings declined annually, while the coverage rate remained relatively stable, with an overall rate of 95.76% (2A). Neonatal G6PD enzyme activity demonstrated a positively skewed distribution. A total of 2,564 cases were initially identified as suspected G6PD deficiency, yielding an initial screening positive rate (SPR) of 11.9‰. Notably, the highest number of positive screening results was recorded in 2024 (2C). Among these, 1,027 cases were confirmed by diagnostic testing, corresponding to an estimated overall birth prevalence of 4.77‰ (2E). Similarly, the number of confirmed cases was also highest in 2024. Given the regional heterogeneity of G6PD deficiency distribution, Newborns from nine counties (cities and districts) were further analyzed. In 2024, 24,850 newborns were screened (2B), with an SPR of 13.3‰ (2D) and a birth prevalence rate of 4.9‰ (2F). The Yueyanglou District exhibited both the highest screening population and the highest birth prevalence rate, although its SPR was comparatively lower.

### 3.2 Relationship between gestational age, birth weight, and G6PD activity

#### 3.2.1 Influence of gestational age and birth weight on G6PD activity

Among 215,137 newborns, the G6PD enzyme activities (median [IQR]) were 7.04 (6.78, 8.12) U/g Hb, 6.78 (5.79, 7.78) U/g Hb, and 6.94 (5.76, 7.90) U/g Hb for the preterm birth, full-term, and post-term pregnancy groups, respectively. G6PD activity followed a skewed distribution. The Kruskal–Wallis H test and Dunn’s *post hoc* test revealed significant differences among the three groups (*H* = 326.2, *p* < 0.0001), with a significant difference between preterm and term groups (*p* < 0.0001). No significant differences were found among the other groups (Table 1).

**TABLE 1:** Comparison of the influence of gestational age on G6PD activity among three groups using the Kruskal–Wallis H test.

Groups	N	G6PD activity (U/g Hb)		Kruskal–Wallis *H* value	*p*	Dunn’s test (*p*)
		Median (IQR)	1%			
<37 W (A)	11,995	7.04 (6.78,8.12)	3.60	326.2	<0.0001	A-B: <0.0001
37–41^+6^ W (B)	202,962	6.78 (5.79,7.78)	3.20			B-C: ns
≥42 W (C)	180	6.94 (5.76,7.90)	4.06			A-C: ns

G6PD activity followed a skewed distribution. The non-parametric test revealed a significant difference among the three groups (*H* = 326.2, *p* < 0.0001). Of note, Dunn’s multiple comparison test identified a pronounced difference between the preterm and term groups (p < 0.0001). No significant differences were observed among the other groups. Median (IQR): median and interquartile range; 1%: 1st percentile; ns: no significance.

When stratified by birth weight, the low weight group exhibited a G6PD activity of 6.90 (5.87, 8.00) U/g Hb (Table 2). The normal birth weight group showed the lowest median G6PD activity (6.78 [5.79, 7.79] U/g Hb), while the macrosomic group had a median activity of 6.94 (5.92, 7.95) U/g Hb. Significant differences among the three groups were identified (*H* = 186.4, p < 0.0001), with Dunn’s test indicating differences between the low weight and normal groups (p < 0.0001), as well as between the macrosomic and normal groups (p < 0.0001). No significant differences were found among the other groups.

**TABLE 2:** Comparison of the influence of newborn birth weight on G6PD activity among three groups using the Kruskal–Wallis H test.

Groups	N	G6PD activity (U/g Hb)		Kruskal–Wallis *H* value	*p*	Dunn’s test (*p*)
		Median (IQR)	1%			
<2,500 g (A)	9,021	6.90 (5.87,8.00)	3.40	186.4	<0.0001	A-B: <0.0001
2,500–3,999 g (B)	194,091	6.78 (5.79,7.79)	3.20			B-C: <0.0001
≥4,000 g (C)	12,025	6.94 (5.92,7.95)	3.48			A-C: ns

G6PD activity also followed a skewed distribution. The non-parametric test revealed a significant difference among the three groups (*H* = 186.4, *p* < 0.0001). Of note, Dunn’s multiple comparison test specified a remarkable difference between the low weight group and the normal group (*p* < 0.0001), as well as between the macrosomic group and the normal group (*p* < 0.0001). No significant differences were detected among the other groups. 1%: 1 percentile ns: no significance.

#### 3.2.2 Gestational age is negatively correlated with G6PD activity

Multiple linear regression analysis was performed to assess the independent effects of gestational age (GA) and birth weight on G6PD activity, with a model constant of 9.935 U/g Hb representing the theoretical baseline (Table 3). Results indicated that G6PD activity decreased by 0.080 U/g Hb per additional week of gestational age (*β* = −0.080, *p* < 0.001), implying an independent and consistent negative association between GA and G6PD activity. Conversely, birth weight did not significantly influence G6PD activity (*β* ≈ 0, *p* = 0.838).

**TABLE 3:** Gestational age is negatively correlated with G6PD activity.

Model	Unstandardized Coefficient		Standardized Coefficient	*t*	*p*
	B	SE	*β*		
(Constant)	9.935	0.094	​	106.162	<0.001
Gestational age	-0.08	0.002	-0.071	-33.201	<0.001
Birth weight	-9.16E-09	0	0	-0.204	0.838

Multiple linear regression analysis was conducted to evaluate the independent effects of gestational age and birth weight on G6PD, activity. Results elucidated that G6PD, activity decreased by 0.080 U/g Hb for each additional gestation age (β = −0.080, p < 0.001). Conversely, birth weight did not significantly contribute to the variation in G6PD, activity (*β* ≈ 0, *p* = 0.838).

### 3.3 Optimization of the cut-off value to 3.2 U/g Hb for male neonates

**FIGURE 3:**
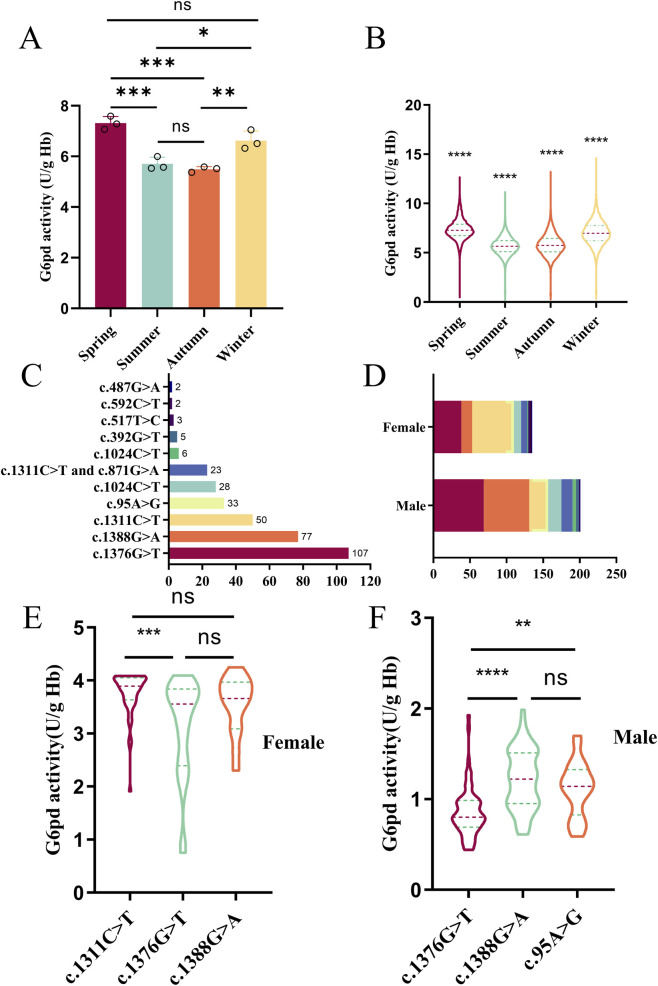
The median G6PD activity and G6PD gene mutation spectrum. **(A)** Comparison of median G6PD activity between seasons. From November 2022 to December 2025, 72,811 newborns were analyzed to evaluate seasonal effects on cut-off values. Newborns were categorized into four groups: spring, summer, autumn, and winter. Median values demonstrated significant differences between the spring–winter and summer–autumn groups. **(B)** Comparison of enzymatic activity distributions across seasons. G6PD activity exhibited a skewed distribution. A Non-Parametric test (Kruskal-Wallis test) was employed to analyze significant differences among groups. **** represents *p* < 0.0001 (between any two groups). **(C)** G6PD gene mutation spectrum and frequency distribution. Genetic analysis confirmed 336 neonates. The six most prevalent mutations were c.1376G>T (31.85%), c.1388G>A (22.92%), c.1311C>T (14.88%), c.95A>G (9.82%), c.1024C>T (8.33%), and c.871G>A (6.85%). **(D)** Comparison of G6PD gene mutation spectrum by sex. Among 201 male cases, the predominant mutant alleles were c.1376G>T, c.1388G>A, and c.95A>G. Among 135 female cases, the predominant variants were c.1311C>T, c.1376G>T, and c.1388G>A. **(E,F)** Comparison of G6PD enzymatic activity among different gene mutations. G6PD activity was higher in females than in males. The c.1376G>T mutation was associated with the most pronounced reduction in enzymatic activity in both sexes, with median activities of 0.8 U/g Hb in males and 3.54 U/g Hb in females. In contrast, the c.1311C>T mutation had minimal impact on G6PD activity in females, with a median value of 3.89 U/g Hb.

**FIGURE 4:**
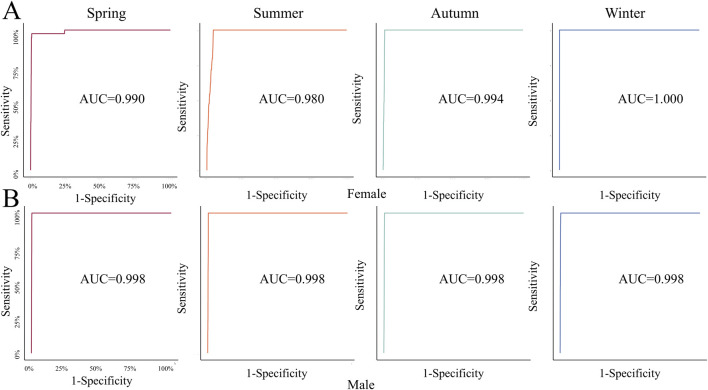
Comparison of ROC curves for G6PD activity between different seasons (**(A)** Female, **(B)** Male). In female neonates, ROC curves demonstrated excellent performance across all seasons, with AUCs of 0.990, 0.980, 0.994, and 1.000, respectively. Despite noticeable differences in AUC values between summer and autumn/winter (Table 4), the optimized cut-off value remained consistent at approximately 4.1 U/g Hb (range: 4.025–4.105 U/g Hb). In male neonates, the cut-off value was 2.6 U/g Hb, and ROC curves demonstrated excellent performance across all seasons, with AUCs of 0.998.

Over the past 3 years (November 2022 to December 2025), 72,811 newborns were evaluated to assess the impact of seasonal factors on screening cut-off values. Newborns were categorized into four groups according to birth season: spring (March–May), summer (June–August), autumn (September–November), and winter (December–February). The median (Figure 3A) and overall distribution (Figure 3B) of G6PD activity across seasons showed significant differences (*p* < 0.0001). Previous data demonstrated gender-related heterogeneity in G6PD activity. Accordingly, cut-off values were set at 2.6 U/g Hb for male neonates and 4.1 U/g Hb for female neonates. Receiver operating characteristic (ROC) curve analyses were subsequently performed for females and males, separately (Figure 4). In female neonates, ROC curves demonstrated excellent diagnostic performance across all seasons, with AUCs of 0.990, 0.980, 0.994, and 1.000, respectively. Although AUC values varied between summer and autumn/winter (Table 4), the optimized seasonal cut-off remained consistent at approximately 4.1 U/g Hb (range: 4.025–4.105 U/g Hb). Applying the uniform cut-off of 4.1 U/g Hb yielded a sensitivity (≥97.4%) and specificity (≥95.2%) (Table 4). In male neonates, with a cut-off value of 2.6 U/g Hb, ROC curves showed optimal performance across all seasons, with an AUC of 0.998 (Figure 4). Sensitivity and specificity were stable in spring, summer, and autumn (Table 5), indicating no need for optimization. However, in winter, the optimal cut-off value was determined to be 3.215 U/g Hb, with an AUC of 0.998, achieving 100% sensitivity, 99.6% specificity, and a Youden index of 0.996 (Table 5), clearly superior to the performance at 2.6 U/g Hb (sensitivity 0.976, specificity 0.996, Youden index 0.952).

**TABLE 4:** Comparison of ROC curves of G6PD activity in female neonates between different seasons.

Season	Cut-off value	AUC	Sensitivity	Specificity	Pairwise comparison	
					Variables	*p*
Spring	4.105	0.99	0.974	0.991	Spring/Summer Spring/Autumn	0.116 0.536
Summer	4.095	0.98	1.000	0.954	Summer/Autumn Summer/Winter	<0.001 <0.001
Autumn	4.105	0.994	1.000	0.989	Autumn/Winter	0.425
Winter	4.025	1.000	1.000	1.000	Winter/Spring	0.130

The cut-off value was established at 4.1 U/g Hb for female neonates. ROC analysis illustrated the excellent performance among all seasons, with AUCs of 0.990, 0.980, 0.994, and 1.000, respectively. Although the AUC value differed noticeably between summer and autumn/winter, the optimized cut-off value remained consistent at approximately 4.1 U/g Hb (range: 4.025–4.105 U/g Hb), yielding a sensitivity (≥97.4%) and specificity (≥95.2%). Therefore, optimization of the cut-off value is not warranted.

**TABLE 5:** Comparison of ROC curves of G6PD activity in male neonates between different seasons.

Season	Cut-off value	AUC	Sensitivity	Specificity	Pairwise comparison	
					Variables	*p*
Spring	2.605	0.998	1	0.997	Spring/Summer Spring/Autumn	0.117 0.212
Summer	2.32	0.998	1	0.996	Summer/Autumn Summer/Winter	0.758 0.146
Autumn	2.625	0.998	1	0.995	Autumn/Winter	0.254
Winter	3.215	0.998	1	0.996	Winter/Spring	0.933

The cut-off value was established at 2.6 U/g Hb for male neonates. Sensitivity and specificity were consistent across spring, summer, and autumn, indicating no need for cut-off value optimization. In winter, however, the optimal cut-off value was 3.215 U/g Hb, with an AUC of 0.998, achieving 100% sensitivity and 99.6% specificity, and the Youden index (0.996). This performance was clearly superior to that at 2.6 U/g Hb (sensitivity = 0.976, specificity = 0.996, Youden index = 0.952).

### 3.4 G6PD gene pathogenic variants analysis

Of the 2,564 screen-positive infants, 1,027 were confirmed to have G6PD deficiency. Prior to 2022, confirmatory diagnosis was routinely performed via the G6PD/6PGD enzymatic ratio assay for 2,144 recalled cases. Since 2022, a total of 420 suspected neonates had undergone recall genetic testing, among whom 336 tested positive, and 84 were ruled out. All genetic data were included in the subsequent analysis. Among them, 201 males were hemizygous, 133 females were heterozygous, and 2 females were homozygous (c.1311C>T), yielding a positive predictive value of 87.14%.

Ten distinct G6PD pathogenic variants were identified. The c.1376G>T variant was the most prevalent, accounting for 31.85% of deficient alleles, followed by c.1388G>A (22.92%), c.1311C>T (14.88%), c.95A>G (9.82%), c.1024C>T (8.33%), and c.871G>A (6.85%) (Figure 3C). Among the 201 male cases, the predominant mutant alleles were c.1376G>T, c.1388G>A, and c.95A>G. Homoplastically, among the 135 female cases, the main variants were c.1311C>T, c.1376G>T, and c.1388G>A (Figure 3D). The results indicated higher G6PD activity in females than in males. The c.1376G>T variant was associated with the greatest reduction in enzyme activity, with median G6PD activities of 0.8 U/g Hb in males and 3.54 U/g Hb in females (Figures 3E,F). In contrast, the c.1311C>T variant had minimal effect on G6PD activity in females, with a median value of 3.89 U/g Hb (Figure 3E).

Given the possibility of false negatives, genetic testing was performed on newborns with negative screening results born in winter to verify the appropriateness of the optimized cut-off value. Nine samples were selected (male: n = 5, female: n = 4) with marginal G6PD activity levels (male: 2.6–3.2 U/g Hb; female: 4.1–4.3 U/g Hb). Four male newborns were hemizygous, and four female newborns were heterozygous, resulting in a genetic mutation rate in borderline samples of 88.89%. Moreover, given suspected analytical bias and incomplete coverage of target loci in routine genetic testing, additional sequencing was performed on the remaining 9 specimens. 3 cases were confirmed, harboring the pathogenic variants of c.406C>T; c.95A>G; and c.1004C>A, accompanied by a hemizygous pathogenic variant involving c.1311C>T and the c. IVS-93T>C site, respectively.

## 4 Discussion

In the study, a total of 215,137 neonates were enrolled. The cut-off values for neonatal screening were optimized according to the gestational age (GA), season of birth, and gender. Concurrently, the spectrum of G6PD gene pathogenic variants in the region was delineated, and the correlation between specific mutations and G6PD enzyme activity was analyzed. The regional pathogenic variant spectrum provides a robust foundation for targeted prevention and control strategies. Notably, the incidence in north Hunan was lower than previously reported rates in Guizhou (Liu et al., 2020), Yunnan (He et al., 2018), and Guilin (Yang et al., 2025) in southern China.

Our findings show that G6PD activity was highest in the preterm birth group and declined toward term, consistent with findings from other studies (Alkaabi et al., 2025; Jiang et al., 2021; Iyer et al., 2024). Indeed, GA is identified as a critical determinant of G6PD activity. Compared with the full-term group, the preterm-birth group demonstrated significantly higher G6PD activity across median and 1st percentile values (3.6 U/g Hb). Multiple linear regression analysis confirmed that GA exerted an independent and consistent negative regulatory effect on G6PD activity, whereas the linear effect of birth weight was negligible. Conventionally, due to the immature development of multiple organs in preterm birth neonates, these infants often exhibit insufficient enzyme activity. Interestingly, this is contrary to the claim in the G6PD deficiency. Previous studies have demonstrated no significant correlation between the percentage of GSH reduction and GA age (Ko et al., 2009). Suggesting that the increased enzyme activity in preterm infants is not primarily due to immature antioxidant mechanisms. A plausible explanation is that the rapid increase in red blood cell levels may contribute to elevated G6PD activity. Mechanistically, preterm infants exhibit higher G6PD levels primarily because they have a markedly higher proportion of young erythrocytes and reticulocytes in circulation. G6PD activity is naturally at its peak in young red blood cells and progressively decays as cells age (Ko et al., 2009; Mesner et al., 2004). Meanwhile, the GA stratified activity values are offered as useful scientific evidence for laboratories and epidemiology, implying that it is necessary to focus on the missed screening of G6PD deficiency in premature infants. Considering the risk of false-negative results, it is recommended that preterm neonates with G6PD activity between 2.6 U/g Hb and 3.6 U/g Hb be recalled for genetic testing if necessary to avoid missed screening.

Given that screening positive rates (SPR) were notably lower in winter and spring compared to summer and autumn, a considerable proportion of neonates with potential partial G6PD deficiency may be missed in winter. Therefore, the optimal cut-off values for G6PD deficiency should be further refined and optimized according to seasonal variations. Yueyang City, located in central China, experiences significant seasonal temperature fluctuations—particularly during summer—which may influence the collection, storage, and transportation of dried blood spots (DBS). The observed seasonal variation in median G6PD activity supports this hypothesis. Consequently, based on seasonal ROC curve analyses, optimal gender-specific cut-off values were established. For female neonates, an optimal cut-off of 4.1 U/g Hb was identified (sensitivity: 0.974, specificity: 0.952, AUC: 0.952), demonstrating excellent diagnostic performance and adequately accounting for seasonal fluctuations in G6PD activity. Similarly, for male neonates born in winter, the initial optimal cut-off was 2.6 U/g Hb (sensitivity: 0.976, specificity: 0.996, AUC: 0.952). When adjusted to 3.215 U/g Hb, sensitivity reached 1.000 with maintained specificity (0.996) and improved AUC (0.998). Considering the possibility of false-negative results, nine negative cases were evaluated through genetic testing, yielding a genetic mutation rate in borderline samples of 88.89%. This indicates that newborns born in winter are more likely to be missed during screening. However, the conclusion might be unreliable due to the small sample size for false-negative verification and the fact that all evaluated newborns were born in winter. Theoretically, all neonates with marginal G6PD activity (male: 2.6–3.2 U/g Hb; female: 4.1–4.3 U/g Hb) were included in the genetic mutation rate in borderline samples analysis, which substantially improves evaluation performance. It is strongly recommended that for female newborns, the optimal cut-off remain at 4.1 U/g Hb, whereas for male newborns in winter, adjusting the cut-off from 2.6 to 3.2 U/g Hb may enhance sensitivity and minimize false-negative results. For other seasons, maintaining the existing cut-offs appears reasonable. This may be explained by the fact that males possess only one X chromosome, rendering G6PD enzyme activity more susceptible to environmental influences such as temperature. G6PD activity may decline during summer and recover in winter (Li et al., 2024).

In recent years, genetic analysis of G6PD deficiency has become a priority in newborn screening programs in China (Zhang et al., 2025; Jiang, 2024). Suspected genetic abnormalities can be further confirmed through sequencing. Our results clearly indicate the presence of additional mutation sites. The prevalence rate is only 3/215,137, which is acceptable. Investigating the distribution characteristics of G6PD pathogenic variants in specific regions can identify asymptomatic G6PD deficiency and prevent severe hyperbilirubinemia and neurologic damage.

Among 336 neonates, the six most prevalent mutations were c.1376G>T (31.85%), c.1388G>A (22.92%), c.1311C>T (14.88%), c.95A>G (9.82%), c.1024C>T (8.33%), and c.871G>A (6.85%). These mutation patterns differ partially from those reported in other regions of China (Jiang, 2024; Fu et al., 2018). For instance, c.487G>A is predominant in Yunnan, c.1388G>A in Guangxi (Fu et al., 2018), and c.1024C>T in Guizhou, further supporting the regional heterogeneity in G6PD deficiency genotypes. Alternatively, the c.1376G>T mutation was associated with a more pronounced reduction in G6PD enzyme activity in both male and female neonates, implying a higher risk of hemolysis. In contrast, the c.1311C>T mutation resulted in the smallest decrease in enzyme activity among females, with a median value of 3.89 U/g Hb. Indeed, neonates carrying the c.1376G>T allele warrant particular attention due to their elevated hemolytic risk. The preventive measures should include family education, strict avoidance of hemolysis-inducing agents, including certain foods and medications (Huang et al., 2024), and close monitoring. Additionally, G6PD activity levels may serve as a preliminary indicator for inferring potential mutations and assessing hemolysis risk, thereby enabling early intervention (Chamchoy et al., 2023). The pathogenesis of G6PD deficiency is well established due to genetic mutations that impair enzyme function (Zhang et al., 2025), with mutation types known to be highly diverse (Nannelli et al., 2023). Undoubtedly, clarifying the correlation between common regional mutations and enzyme activity levels is crucial for clinical counseling, diagnosis, and management of G6PD deficiency.

Notably, this study has several limitations. First, considering the possibility of false negatives, genetic testing was performed on newborns with negative screening results who were born in winter to evaluate the appropriateness of the optimized cut-off value. This finding suggests that newborns born in winter are more likely to be missed during screening. However, the limited number of confirmed false negatives might lead to an overestimation of diagnostic accuracy in the ROC curve analysis. Owing to the limited number of borderline samples available for verifying genetic mutation rates and the seasonal bias that all enrolled neonates were delivered in winter, our study has inherent limitations. We plan to improve upon these drawbacks in future research, with a special focus on male infants. Second, the genetic assay was limited to 10 known hotspot mutations, inevitably missing rare or novel variants and potentially contributing to false-negative results.

In conclusion, this large-scale newborn screening study conducted in Yueyang City identified gestational age as a key factor influencing G6PD activity. Simultaneously, for male neonates born in winter, a revised cut-off value of 3.2 U/g Hb is recommended to reduce false-negative rates. This study primarily explored the epidemiological distribution and pathogenic variant spectrum of G6PD deficiency in the Yueyang region, aiming to provide evidence for the prevention of severe hyperbilirubinemia and subsequent neurological damage. It provides valuable data for genetic counseling and early clinical diagnosis. Early screening and detection, intensified follow-up management, and the effective implementation of the prenatal and postnatal care policy constitute the core of our work. Meanwhile, detailed precautionary guidance remains extremely important, including dietary restrictions and a list of restricted-use medications, underscoring the pivotal role of newborn screening for G6PD deficiency in disease management.

## Data Availability

The datasets presented in this study can be found in online repositories. The names of the repository/repositories and accession number(s) can be found in the article/supplementary material.
