# Supplementary material for: Screening of glucose-6-phosphate dehydrogenase deficiency in a cohort of 215,137 newborns: an epidemiological and pathogenic variant spectrum study in Yueyang, China
Source: Front Genet. 2026 Jun 25;17:1810076. doi: 10.3389/fgene.2026.1810076 (PMC13345595; doi:10.3389/fgene.2026.1810076)
Supplement: Supplementary file 1 [file DataSheet1.zip › 3-Table 3.DOCX]

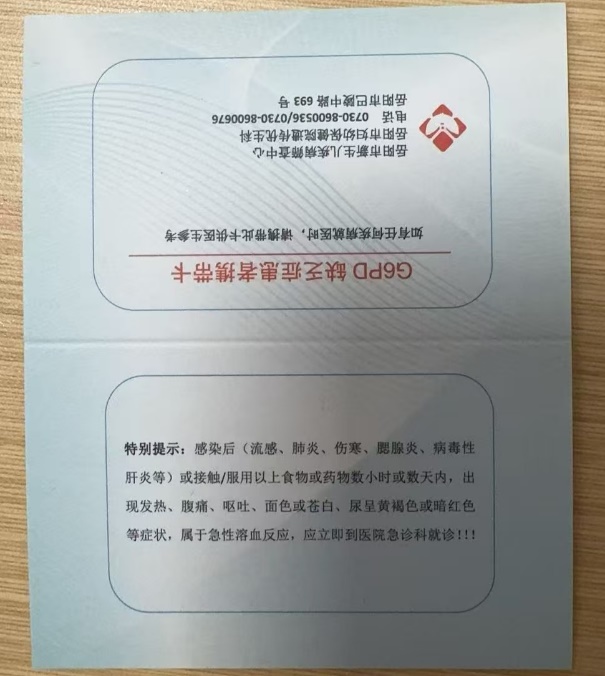

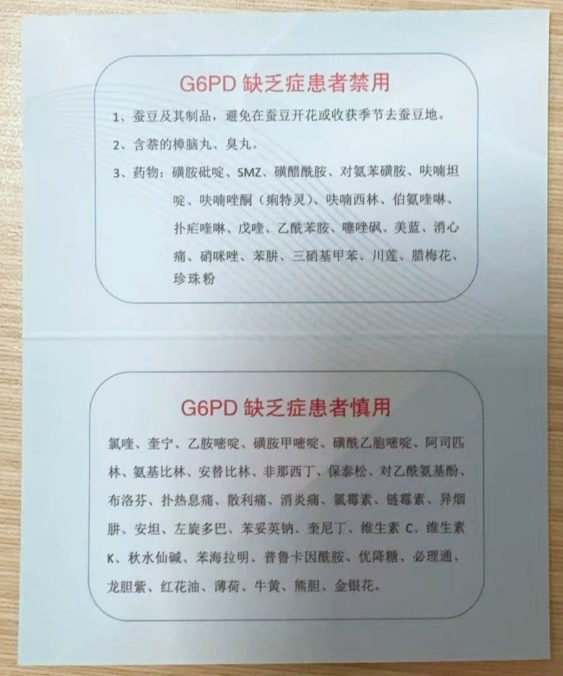


Figure | The card for a patient with G6PD deficiency. Upon confirmation, the patient was referred for outpatient services. Cases were recorded in the case management ledger. Timely follow-up and detailed precautionary guidance were provided, including a list of dietary restrictions and medications to be used with caution.

**Prohibited List**

1. Broad beans and their products. Avoid going to the broad bean fields during the flowering or harvesting seasons.

2. Toluene-containing camphor ball.

3. Medications: Sulfapyridine, SMZ, Sulfacetamide, Sulfanilamide, etc.

**Cautionary List**

Chloroquine, Quinine, Pyrimethamine, Sulfamerazine, Aspirin, Aminopyrine, Ibuprofen, Vitamin C, Chloramphenicol, Streptomycin, Isoniazid, etc.
